# Treatment-free remission following frontline nilotinib in patients with chronic phase chronic myeloid leukemia: 5-year update of the ENESTfreedom trial

**DOI:** 10.1038/s41375-021-01205-5

**Published:** 2021-03-11

**Authors:** Jerald P. Radich, Andreas Hochhaus, Tamás Masszi, Andrzej Hellmann, Jesper Stentoft, María Teresa Gómez Casares, J. Valentín García-Gutiérrez, Eibhlin Conneally, Philipp D. le Coutre, Norbert Gattermann, Bruno Martino, Susanne Saussele, Francis J. Giles, David M. Ross, Paola Aimone, Sai Li, Ksenia Titorenko, Giuseppe Saglio

**Affiliations:** 1 Clinical Research Division, Fred Hutchinson Cancer Research Center, Seattle, WA USA; 2 Abteilung Hämatologie/Onkologie, Universitätsklinikum Jena, Jena, Germany; 3 Semmelweis University, Budapest, Hungary; 4 Medical University of Gdańsk, Gdańsk, Poland; 5 Aarhus University Hospital, Aarhus, Denmark; 6 Hospital Universitario de Gran Canaria Dr Negrín, Las Palmas de Gran Canaria, Spain; 7 Hospital Universitario Ramón y Cajal, IRYCIS, Madrid, Spain; 8 St James’s Hospital, Dublin, Ireland; 9 Charité-Universitätsmedizin Berlin, Berlin, Germany; 10 Universitätsklinikum Düsseldorf, Düsseldorf, Germany; 11 Azienda Ospedaliera Bianchi Melacrino Morelli, Reggio Calabria, Italy; 12 III. Med. Klinik, Medizinische Fakultät Mannheim der Universität Heidelberg, Mannheim, Germany; 13 Developmental Therapeutics Consortium, Chicago, IL USA; 14 Division of Haematology, SA Pathology, Royal Adelaide Hospital, Adelaide, Australia; 15 Novartis Pharma AG, Basel, Switzerland; 16 Novartis Pharmaceuticals Corporation, Moscow, Russian Federation; 17 University of Turin, Orbassano, Italy

**Keywords:** Phase II trials, Chronic myeloid leukaemia

## Abstract

The ENESTfreedom trial assessed the feasibility of treatment-free remission (TFR) in patients with chronic myeloid leukemia in chronic phase (CML-CP) following frontline nilotinib treatment. Results for long-term outcomes after a 5-year follow-up are presented herein. Patients who had received ≥2 years of frontline nilotinib therapy and achieved MR^4.5^ underwent a 1-year nilotinib treatment consolidation phase before attempting TFR. At the 5-year data cut-off, 81/190 patients entering the TFR phase (42.6%) were still in TFR, with 76 (40.0%) in MR^4.5^. Patients who lost major molecular response (MMR) entered a treatment re-initiation phase; 90/91 patients entering this phase (98.9%) regained MMR and 84/91 patients (92.3%) regained MR^4.5^. The Kaplan–Meier estimated treatment-free survival rate at 5 years was 48.2%. No disease progression or CML-related deaths were reported. Whereas the incidence of adverse events (AEs) declined from 96 weeks following the start of TFR, an increase in AE frequency was observed for patients in the treatment re-initiation phase. Low Sokal risk score, *BCR-ABL1*^IS^ levels at 48 weeks of TFR and stable MR^4.5^ response for the first year of TFR were associated with higher TFR rates. Overall, these results support the efficacy and safety of attempting TFR following upfront nilotinib therapy of >3 years in patients with CML-CP.

## Introduction

Outcomes for patients with chronic myeloid leukemia (CML) have improved dramatically over the past years, with patients in Western nations having a life expectancy similar to those of age-matched individuals in the general population [1–6]. This success has shifted the focus of CML treatment from survival to improved quality of life and reduction of adverse events (AEs) [7–9]. Long-term treatment with tyrosine kinase inhibitors (TKIs) can lead to cardiovascular and musculoskeletal toxicity and, among other events, even minor AEs such as fatigue may have a substantial impact on the quality of life when persisting for long periods of time [7, 10]. Life-long treatment also represents a major cost burden on healthcare systems, particularly considering the increase in life expectancy, and may result in non-adherence to treatment for those patients who cannot afford it [9, 11].

For these reasons, whereas previously life-long treatment with TKIs was recommended, current guidelines for CML management support the safety of the treatment-free remission (TFR) approach for certain patients and provide guidance on patient selection and monitoring [5, 12, 13]. Previous studies in patients with CML have shown that TFR rates range from 43 to 72% for at least 6 months and up to 44 months after stopping TKI treatment [14–22]. A recent meta-analysis of 1,601 patients showed that the estimated weighted mean incidence of loss of major molecular response was 41% at 24 months after treatment discontinuation, with 82% of molecular relapses occurring in the first 6 months [23].

Despite the apparent success of TFR, there is a need for more research on treatment discontinuation in order to identify those patients who are most likely to benefit from stopping treatment [9]. Another issue of concern is that patients in TFR can present a different spectrum of AEs compared with those receiving treatment: for example, a syndrome similar to polymyalgia, presenting with musculoskeletal and/or joint pain weeks after stopping TKI treatment, has been described in 20–30% of patients and is known as TKI withdrawal syndrome [12]. However, in most cases this syndrome is self-limited, unlike AEs reported while on therapy.

ENESTfreedom is the first study to assess TFR in patients with chronic phase CML (CML-CP) achieving deep molecular response (DMR) following frontline nilotinib treatment for >3 years [13]. In the primary analysis, 51.6% of patients were still in TFR 48 weeks after stopping treatment, remaining in major molecular response (MMR; *BCR-ABL1* ≤ 0.1% on the International Scale [*BCR-ABL1*^IS^]) or better [13]. At 96 weeks after the start of the TFR phase, 48.9% of patients remained in MMR [24]. Among the patients who experienced loss of MMR, 98.9% regained MMR and 92.0% regained MR^4.5^ (defined as *BCR-ABL1*^IS^ ≤ 0.0032%) following nilotinib treatment re-initiation [24]. The frequency of most AEs decreased in a time-dependent manner during the TFR phase [13, 24]. It should be noted that in this study previous exposure to nilotinib was ~3.5 years, which is shorter than that reported in several other TKI studies [16, 17, 19, 20, 25].

To evaluate the long-term outcomes of patients in TFR following frontline nilotinib treatment, we present here an updated analysis of the ENESTfreedom study after a 5-year follow-up period.

## Materials and methods

### Study design and patients

**Fig. 1:**
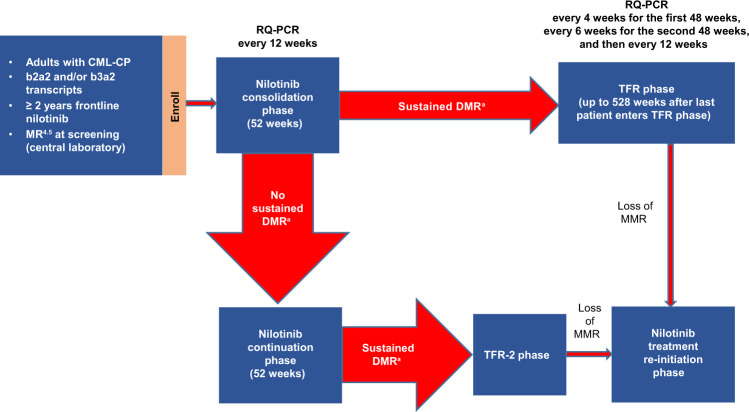
ENESTfreedom study design. ^a^ DMR was defined as the following (in the last 4 quarterly RQ-PCR assessments): MR^4.5^ in the last assessment, ≤2 assessments between MR^4^ and MR^4.5^, and no assessment worse than MR^4^. CML-CP, chronic myeloid leukemia in chronic phase; DMR, deep molecular response; MMR, major molecular response (*BCR-ABL1* on the International Scale [*BCR-ABL1*^IS^] ≤ 0.1%), MR^4^, *BCR-ABL1*^IS^ ≤ 0.01%; MR^4.5^, *BCR-ABL1*^IS^ ≤ 0.0032%; RQ-PCR, real-time quantitative polymerase chain reaction; TFR, treatment-free remission.

Study design and eligibility criteria for ENESTfreedom (NCT01784068)—a single-arm, phase 2 study—have been previously described [13]. Briefly, patients (aged ≥ 18 years) with Philadelphia chromosome-positive CML-CP who had received ≥2 years of frontline nilotinib therapy and achieved MR^4.5^ were eligible to enroll and entered a 52-week consolidation phase in which they continued nilotinib treatment (Fig. 1). During this phase, molecular responses were monitored every 12 weeks by real-time quantitative polymerase chain reaction (RQ-PCR). Of note, detection of *BCR-ABL1*^IS^ levels prior to study entry was performed by external laboratories, while confirmatory testing for trial eligibility and subsequent molecular testing was performed at a study-centralized laboratory.

Patients who achieved sustained DMR (defined as MR^4.5^ in the most recent assessment, ≤2 assessments between MR^4^ [*BCR-ABL1*^IS^ ≤ 0.01%] and MR^4.5^, and no assessment worse than MR^4^ in the last 4 quarterly RQ-PCR assessments) during the consolidation phase stopped nilotinib treatment and entered the TFR phase, in which they were monitored by RQ-PCR every 4 weeks during the first 48 weeks, every 6 weeks during the next 48 weeks, and every 12 weeks thereafter. Monitoring is scheduled to continue for up to 10 years after the last patient enters the TFR phase.

Nilotinib treatment was re-initiated upon any single assessment showing loss of MMR (defined as *BCR-ABL1*^IS^ > 0.1%). Patients in the nilotinib treatment re-initiation phase were monitored every 4 weeks for the first 24 weeks after MMR loss and every 12 weeks thereafter to assess potential regaining of molecular response.

### Updated and additional analyses

We report here updated results based on a data cut-off date of 3^rd^ February 2020, at which time all patients who entered the TFR phase had completed 5 years of TFR, switched to the re-initiation phase, or discontinued from the study. The duration of follow-up in the TFR phase (in weeks) was calculated as = (end date of TFR phase – start date of TFR phase + 1 day)/7. For patients who left TFR before the end of the TFR phase, the end date of TFR phase is the earliest occurrence of loss of MMR or any other reason for discontinuation of the TFR phase. For patients ongoing in the TFR phase at the time of data cut-off, the cut-off date is used as end date of TFR phase.

TFR rates were calculated as percentages with an exact 95% Clopper-Pearson confidence interval. Treatment-free survival (TFS)—defined as the time from the start of TFR to the earliest occurrence of any of these events: loss of MMR, nilotinib treatment re-initiation due to any reason, progression to accelerated phase/blast crisis (AP/BC), or death from any cause—was estimated using the Kaplan–Meier method. For patients without any such event, TFS was censored at the date of the last assessment. A sensitivity analysis was conducted considering discontinuation from TFR phase due to any reason (in addition to the events described above) as an event.

Progression-free survival (PFS)—defined as the time from the start of the TFR phase to the earliest occurrence of either progression to AP/BC or death due to any cause—and overall survival (OS)—defined as the time from the start of the TFR phase to death due to any cause—were also estimated using the Kaplan–Meier method. Rates of response re-achievement were calculated as cumulative incidence.

To investigate factors potentially associated with successful TFR at 5 years, we divided all patients attempting TFR into subgroups according to their Sokal risk score at diagnosis, MR^4.5^ rate at 48 weeks, and MR^4.5^ stability during the first 48 weeks of the TFR phase. TFR rates were calculated at 5 years for each subgroup.

Safety data included a description of AEs reported during the consolidation phase and the first, second, third, fourth, and fifth 48 weeks of the TFR phase for those patients remaining in the TFR phase for longer than 3.7 years, as well as overall AEs for patients in the treatment re-initiation phase. A description of AEs reported during the consolidation phase and the first, second, and third 48 weeks of the TFR phase is also presented for all patients who entered the TFR phase. Musculoskeletal pain-related AEs were grouped, including events reported by the preferred terms of myalgia, arthralgia, bone pain, spinal pain, pain in extremity, and musculoskeletal pain. Time to first musculoskeletal pain event and duration of first musculoskeletal pain event among patients entering the TFR phase were estimated using the Kaplan–Meier method. AEs were assessed as per the Common Terminology Criteria for Adverse Events version 4.03.

### Ethics

ENESTfreedom was designed and conducted in accordance with the ethical principles of the Declaration of Helsinki, the International Conference on Harmonisation (ICH) Harmonized Tripartite Guidelines for Good Clinical Practice and local laws and regulations. Written informed consent was provided by all patients before any study procedures. The study protocol and its amendments were reviewed and approved by an independent ethics committee or institutional review board for each study center.

## Results

### Patient disposition

Patient demographics, clinical staging, and prior treatment have been previously reported [13, 24]. The median duration of nilotinib exposure was ~1 year (52.1 weeks, range 8.6–57.6 weeks) for patients in the consolidation phase and 4.3 years (224.0 weeks, range 5.0–287.7 weeks) for patients in the treatment re-initiation phase. The median duration of patient follow-up in the TFR phase at the 5-year data cut-off date was 1.5 years (75.9 weeks, range 7.6–303.0 weeks).

**Fig. 2:**
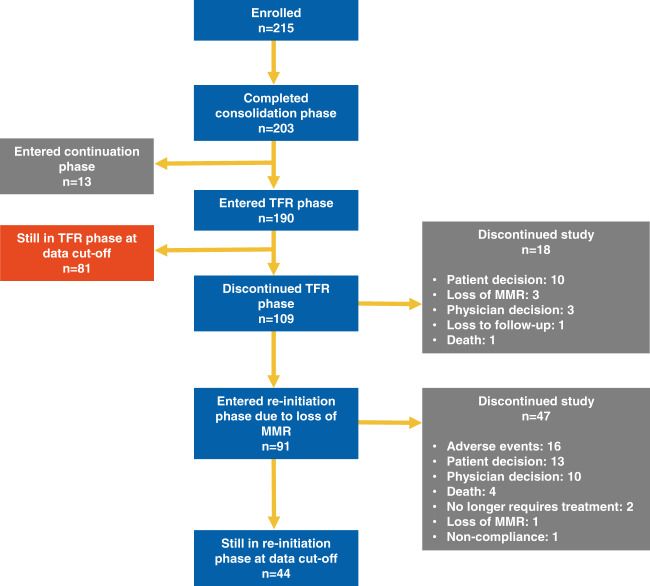
Patient flow and disposition at the 5-year cut-off date. MMR, major molecular response; TFR. treatment-free remission.

Of the originally enrolled 215 patients, 203 completed the consolidation phase. Thirteen of these patients did not achieve stable DMR after the consolidation phase and continued their treatment with nilotinib for a further 52 weeks. Of the 190 patients who entered the TFR phase, 81 (42.6%) were still in TFR at data cut-off (Fig. 2). Among the 109 patients who discontinued the TFR phase, 91 (83.5%) entered the treatment re-initiation phase due to loss of MMR. Nearly half of all patients who re-initiated nilotinib treatment were still in the re-initiation phase at data cut-off (44/91, 48.4%), while the remaining 47 patients discontinued the study for various reasons. The most common reason for study discontinuation (23/47) was physician or patient decision. Death was the reason for study discontinuation for four patients, two patients no longer required treatment, and one discontinued due to loss of MMR, while one patient discontinued treatment because of non-compliance. Out of the 47 patients who discontinued the study, 16 did so due to AEs, which included vascular disorders (six patients), cardiac disorders (four patients), other neoplasms (four patients), and musculoskeletal and connective tissue disorders (three patients). It should be noted that some patients experienced more than one event.

At the 5-year cut-off, 79 out of the 190 patients (41.6%) who entered the TFR phase remained in MMR or better without nilotinib treatment, with 76 (40.0%) remaining in MR^4.5^. For two out of the 81 patients in TFR phase at 5 years, RQ-PCR assessment was not available at this time point and hence their response was not considered for 5-year data analysis. Four patients with MR^4.5^ at 5 years had a temporary loss of MR^4.5^ (at least one *BCR-ABL1*^IS^ transcript level >0.0032%) before the 5-year cut-off, meaning that 72/190 (37.9%) patients remained continuously in MR^4.5^ for the first 5 years after the start of TFR.

At the 5-year cut-off, a total of 94 patients had discontinued the TFR phase due to loss of MMR, while 15 patients had discontinued the study for other reasons (10 withdrew consent, 3 due to physician decision, 1 death and 1 lost to follow-up). Out of these 94 patients, 88 lost MMR during the first 48 weeks, with three additional patients each experiencing loss of MMR in the second and third 48 weeks after treatment discontinuation. Four of the six patients who lost MMR after 48 weeks in TFR had lost MR^4.5^ during the first 48 weeks, while another had unstable MR^4.5^ during the same period; the remaining patient had stable MR^4.5^at 48 weeks.

**Fig. 3:**
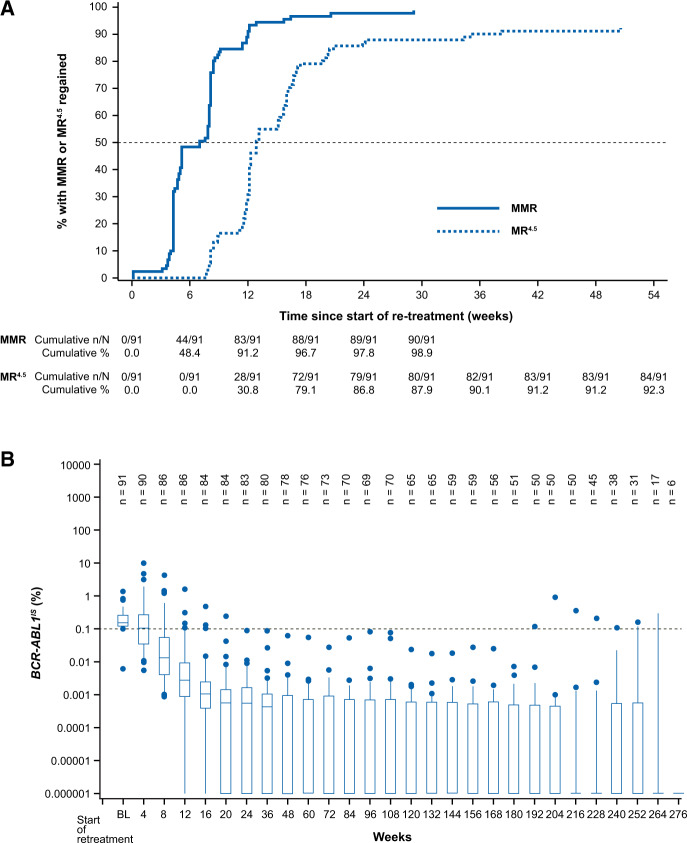
Regain of molecular response following treatment re-initiation. **A** Cumulative MMR and MR^4.5^ rates following treatment re-initiation. **B** *BCR-ABL1*^IS^ transcript levels following treatment re-initiation. Transcript levels were monitored every 12 weeks during the treatment re-initiation phase. Levels of *BCR-ABL1* mRNA transcripts were determined by RQ-PCR testing of peripheral blood and analyzed using a validated RQ-PCR technology standardized to the IS. *BCR-ABL1* transcript levels are expressed in relation to the *ABL1* transcript as an endogenous reference. The *BCR-ABL1*/*ABL1* ratio was calculated and converted to the IS by applying a 1.0 Conversion Factor. Plot shows boxes (25th-75th percentiles) with the median as a horizontal line. Whiskers (vertical lines) extend to the 10th–90th percentiles. Dots show outliers. IS international standard, MMR major molecular response (*BCR-ABL1*^IS^ ≤ 0.1%); MR^4.5^, *BCR-ABL1*^IS^ ≤ 0.0032%; RQ-PCR, real-time quantitative polymerase chain reaction.

Of the 91 patients who entered the treatment re-initiation phase, 90 (98.9%) regained MMR, most of them (91.2%) within the first 12 weeks of re-starting nilotinib. Of those 91 patients, 84 (92.3%) regained MR^4.5^ (Fig. 3A). Of the six patients who regained MMR but not MR^4.5^, one patient was still on treatment at the time of data cut-off, and five patients were discontinued from the study (two patients discontinued due to AEs, one patient due to physician decision, one patient due to patient decision, and one patient due to lack of efficacy). The median *BCR-ABL1*^IS^ transcript level at the start of the treatment re-initiation phase was 0.156% (range, 0.006-1.388). *BCR-ABL1*^IS^ transcript levels decreased swiftly following re-initiation of nilotinib treatment (Fig. 3B). The time by which 50% of all retreated patients regained MMR was 7 weeks, and the equivalent time for MR^4.5^ was 12.9 weeks.

### Long-term outcomes

**Fig. 4:**
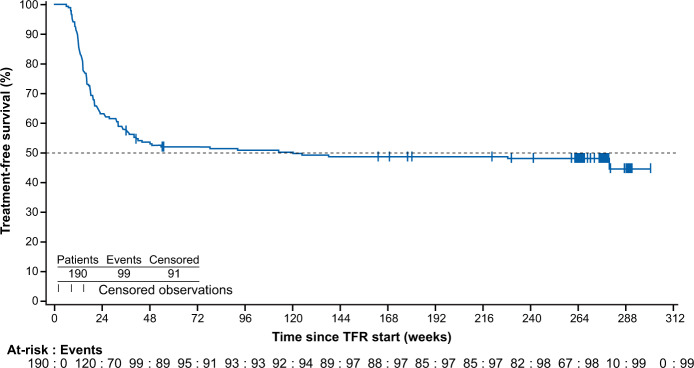
Kaplan–Meier estimate of TFS for all patients who entered the TFR phase. TFS was defined as the time from the date of start of TFR to the date of the earliest occurrence of the following events up to the end of TFR phase: loss of MMR, death due to any cause, progression to AP/BC and re-initiation of treatment due to any cause in the study. AP/BC, accelerated phase/blast phase; MMR, major molecular response; TFR, treatment-free remission; TFS, treatment-free survival.

The TFS rate at 5 years was 48.2% (95% confidence interval [CI] 40.9, 55.1), and the Kaplan–Meier estimated median TFS was 2.3 years (120.1 weeks; 95% CI 36.9, not estimable) (Fig. 4). The curve approached a plateau at ~48 weeks, with little change observed after that, due to the rarity of disease relapse after this time. A sensitivity analysis of TFS was also carried out, assessing discontinuation from TFR phase by the cut-off date due to any reason in addition to those considered to evaluate TFS (loss of MMR, death due to any cause, progression to AP/BC) in order to assess the impact of these additional events on TFS. This analysis was carried out because the majority of later events are due to study discontinuation rather than loss of MMR. In this analysis, an additional 11 patients were considered to have had an event (all 11 patients discontinued the study: 9 due to patient decision, 1 due to physician decision, and 1 was lost to follow-up); due to the small number of additional events, the results appear quite similar to those of the main analysis, with a TFS rate of 42.6% (95% CI 35.5, 49.5) and a Kaplan–Meier estimated median TFS of 1.4 years (74.6 weeks; 95% CI 36.0, 241.1).

### Predictive factors for TFR

Patients with low Sokal risk score at diagnosis had a TFR rate at 5 years of 50.8%, compared with 38.0% for patients with intermediate-risk score and 27.6% for patients with high-risk score (Table 1). A longer history of nilotinib exposure and longer MR^4.5^ response was also associated with higher TFR rates.

**Table 1:** TFR rates in patient subgroups.

TFR rate *n*/*N* (% [95% CI])	TFR (*n* = 190) 5 years
Sokal risk score at diagnosis^a^	
Low	32/63 (50.8 [37.9, 63.6])
Intermediate	19/50 (38.0 [24.7, 52.8])
High	8/29 (27.6 [12.7, 47.2])
Missing	20/48 (41.7 [27.6, 56.8])
Response at week 48 of TFR phase^b^	
MR^4.5^	74/86 (86.0) [76.9, 92.6])
At least MR^4^ but not MR^4.5^	1/3 (33.3 [0.8, 90.6])
At least MMR but not MR^4^	1/5 (20.0 [0.5, 71.6])
Stability of response^b^	
Patients with all RQ-PCR values during the first 48 weeks of TFR phase in MR^4.5^	72/83 (86.7 [77.5, 93.2])
Patients with at least one RQ-PCR value during the first 48 weeks of TFR phase between MR^4.5^ and MR^4^	4/11 (36.3 [10.9, 69.2])
Time since first MR^4.5^ until TFR entry (months)^c^	
<median (30.4)	34/95 (35.8 [26.2, 46.3])
≥median (30.4)	45/95 (47.4 [37.0, 57.9])
Duration of nilotinib treatment prior to TFR entry (months)^c^	
<median (43.5)	34/97 (35.1 [25.6, 45.4])
≥median (43.5)	45/93 (48.4 [37.9, 59.0])

CI, confidence interval; MR^4^, 0.0032% <*BCR-ABL1*^IS^ ≤ 0.01%; MR^4.5^, *BCR-ABL1*^IS^ ≤ 0.0032%); MMR, 0.01% <*BCR-ABL1*^IS^ ≤ 0.1%; RQ-PCR, real-time quantitative polymerase chain reaction; TFR, treatment-free remission.

^a^Sokal risk score was available for two additional patients for this analysis, compared with the primary analysis. This is due to data collection on Sokal score not being planned in the original protocol, resulting in delayed score data collection to the point it was not available for a significant proportion of patients at the time of primary analysis.

^b^Patients who discontinued TFR phase, re-initiated nilotinib or failed TFR by week 48, or had a missing *BCR-ABL1*^IS^ value at 48 weeks were excluded from this analysis.

^c^Detection of *BCR-ABL1*^IS^ levels prior to study entry was performed by external laboratories and not in the study-centralized laboratory.

The TFR rate at 5 years was higher for patients who had remained in MR^4.5^ at week 48 of the TFR phase than for patients who had not (86.0% for patients in MR^4.5^ vs 33.3% for patients in MR^4^ and 20.0% for patients in MMR at week 48 of the TFR phase). The stability of the response was also important for TFR rates at 5 years. Patients with stable MR^4.5^ during the first 48 weeks of the TFR phase had a TFR rate of 86.7%, compared with 36.4% for patients with at least one RQ-PCR value showing loss of MR^4.5^ during the first 48 weeks of TFR.

### Safety

#### TFR phase

86.0% of patients who entered the consolidation phase reported AEs of any grade. This percentage decreased to 79.1% during the first 48 weeks of TFR, 70.9% during the second 48 weeks, 50.0% during the third 48 weeks, and 57.0% during the fourth and fifth 48 weeks of TFR. Grade 3/4 AEs were reported by 14.0% of patients during the first 48 weeks of TFR, 8.1% during the second 48 weeks, 5.8% during the third 48 weeks, 16.3% during the fourth 48 weeks and 8.1% during the fifth 48 weeks of TFR, compared with 12.8% during the consolidation phase.

AEs of special interest at different time points following the start of the TFR phase in the subset of 86 patients remaining in TFR > 3.7 years are shown in Table 2. During the first 48-week period of the TFR phase, 47.7% of patients reported AEs of special interest of any grade; this proportion decreased to 22.1% during the second 48-week period, 7.0% during the third 48-week period, 15.1% during the fourth 48-week period and 10.5% during the fifth 48-week period of the TFR phase, compared with 31.4% during the consolidation phase. The most frequent AEs involved musculoskeletal pain, which increased in frequency from 16.3% of patients during the consolidation phase to 41.9% of patients in the first 48 weeks of TFR. This percentage then decreased to 9.3, 3.5, 5.8, and 4.7% over the second, third, fourth, and fifth 48-week periods of TFR. The Kaplan–Meier estimated median of time to first musculoskeletal pain event was not estimable, whereas the cumulative assessment of loss of musculoskeletal symptoms was 24.1 weeks (95% CI 14.1, 76.1) (Supplementary Fig. 1). Cardiovascular events (CVEs, any grade) were reported by 4.7% of patients during the consolidation phase (for patients remaining in TFR for more than 3.7 years); this percentage decreased to 2.3% in the first 48 weeks following the start of the TFR phase and remained low for the duration of TFR. Among all of the patients who entered the TFR phase (*N* = 190), CVEs were reported in five patients (2.6%) in the first 48 weeks of the TFR phase, one patient (0.5%) in the second, two patients (1.1%) in the third, no patients in the fourth and two patients (1.1%) in the fifth 48 weeks of the TFR phase. For one of the patients, the CVE was first reported during the consolidation phase and continued into the TFR phase.

**Table 2:** AEs of special interest during the TFR phase.

	Consolidation phase *N* = 86	First 48 weeks of TFR *N* = 86	Second 48 weeks of TFR *N* = 86	Third 48 weeks of TFR *N* = 86	Fourth 48 weeks of TFR *N* = 86	Fifth 48 weeks of TFR *N* = 86
Adverse event of special interest group	All grades, n (%)	All grades, n (%)	All grades, n (%)	All grades, n (%)	All grades, n (%)	All grades, n (%)
Total	27 (31.4)	41 (47.7)	19 (22.1)	6 (7.0)	13 (15.1)	9 (10.5)
Blood cholesterol increased	3 (3.5)	4 (4.7)	1 (1.2)	1 (1.2)	1 (1.2)	0
Blood glucose increased	0	1 (1.2)	2 (2.3)	1 (1.2)	1 (1.2)	1 (1.2)
Cardiac failure	0	1 (1.2)	0	0	0	0
Cardiovascular events	4 (4.7)	2 (2.3)	1 (1.2)	2 (2.3)	0	2 (2.3)
Ischemic cerebrovascular events	1 (1.2)	1 (1.2)	0	0	0	0
Ischemic heart disease	1 (1.2)	0	1 (1.2)	1 (1.2)	0	2 (2.3)
Peripheral arterial occlusive disease	1 (1.2)	1 (1.2)	0	1 (1.2)	0	0
Fluid retention	3 (3.5)	3 (3.5)	4 (4.7)	0	4 (4.7)	3 (3.5)
Edema and other fluid retentions	2 (2.3)	3 (3.5)	4 (4.7)	0	4 (4.7)	3 (3.5)
Severe	1 (1.2)	0	0	0	0	0
Hepatotoxicity	2 (2.3)	2 (2.3)	1 (1.2)	0	2 (2.3)	2 (2.3)
Drug-induced liver injury	0	1 (1.2)	1 (1.2)	0	0	0
Total transaminase activity and bilirubin elevations	2 (2.3)	1 (1.2)	0	0	2 (2.3)	2 (2.3)
Musculoskeletal pain	14 (16.3)	36 (41.9)	8 (9.3)	3 (3.5)	5 (5.8)	4 (4.7)
Myelosuppression (Thrombocytopenia)	1 (1.2)	0	0	0	0	0
Pancreatitis, lipase, and amylase elevations	1 (1.2)	0	0	0	0	0
Rash	5 (5.8)	0	2 (2.3)	0	2 (2.3)	0
Significant bleeding	0	0	1 (1.2)	0	0	0
Gastrointestinal hemorrhage	0	0	1 (1.2)	0	0	0
CNS hemorrhage	0	0	0	0	0	0

For patients remaining in TFR > 3.7 years.

AE, adverse event; CNS, central nervous system; TFR, treatment-free remission.

#### Re-initiation phase

The most frequent all-grade AEs of special interest reported during the treatment re-initiation phase were musculoskeletal pain (19.8%), increase in blood cholesterol (18.7%), and CVEs (17.6%) (Table 3). The most frequent CVE reported was ischemic heart disease (8.8% of patients). Overall, there was a considerable increase in all-grade clinically notable AEs in the treatment re-initiation phase compared with the consolidation phase; this is likely due to increased duration of exposure to nilotinib and is consistent with the expected safety profile of nilotinib with a longer treatment duration (Supplementary Table 1).

**Table 3:** AEs of special interest during consolidation and treatment re-initiation phases.

Adverse event of special interest group	Consolidation phase *N* = 215, n (%)	Re-initiation phase *N* = 91, n (%)
Blood cholesterol increased	11 (5.1)	17 (18.7)
Blood glucose increased	3 (1.4)	11 (12.1)
Cardiac failure	0	0
Cardiovascular events	11 (5.1)	16 (17.6)
Ischemic cerebrovascular events	3 (1.4)	3 (3.3)
Ischemic heart disease	3 (1.4)	8 (8.8)
Peripheral arterial occlusive disease	4 (1.9)	4 (4.4)
Fluid retention	4 (1.9)	6 (6.6)
Edema and other fluid retentions	3 (1.4)	4 (4.4)
Severe	1 (0.5)	2 (2.2)
Hepatotoxicity	11 (5.1)	12 (13.2)
Drug-induced liver injury	0	1 (1.1)
Total transaminase activity and bilirubin elevations	11 (5.1)	12 (13.2)
Musculoskeletal pain	33 (15.3)	18 (19.8)
Myelosuppression (Thrombocytopenia)	2 (0.9)	0
Pancreatitis, lipase, and amylase elevations	3 (1.4)	1 (1.1)
QT prolongation	4 (1.9)	4 (4.4)
Rash	8 (3.7)	7 (7.7)
Renal events	0	4 (4.4)
Significant bleeding	2 (0.9)	2 (2.2)
Gastrointestinal hemorrhage	2 (0.9)	2 (2.2)
CNS hemorrhage	0	0

AE, adverse event; CNS, central nervous system.

#### Deaths and disease progression

A total of ten deaths were recorded by the data cut-off date (Table 4) Two deaths occurred during the consolidation phase (one cardiac arrest and one suicide), one during the TFR phase (of unknown cause), four deaths in the re-initiation phase (acute myocardial infarction, hepatobiliary cancer, respiratory failure, and unknown cause) and three during post-treatment follow-up, >30 days after the end of treatment (mesothelioma, transitional cell cancer of the renal pelvis and ureter, and unknown cause). Two new deaths were recorded since the 96-week update, one during the treatment re-initiation phase (hepatobiliary cancer) and one during the post-treatment follow-up (unknown cause). No disease progressions to AP/BC were recorded by the data cut-off date. The Kaplan–Meier estimates at 5 years were 95.6% (95% CI 91.5, 97.8) for PFS and 95.7% (95% CI 91.5, 97.8) for OS.

**Table 4:** Deaths reported by study phase.

	Consolidation phase *N* = 215	TFR phase *N* = 190	Re-initiation phase *N* = 91	Post treatment follow-up	All
Primary cause of death - preferred term	n (%)	n (%)	n (%)	n	n
Total	2 (0.9)	1 (0.5)	4 (4.4)	3	10
Unknown cause	0	1 (0.5)	1 (1.1)	1^a,b^	3
Other cancers	0	0	0	2^a^	2
Acute myocardial infarction	0	0	1 (1.1)	0	1
Cardiac arrest	1 (0.5)	0	0	0	1
Suicide	1 (0.5)	0	0	0	1
Hepatobiliary cancer	0	0	1 (1.1)^b^	0	1
Respiratory failure	0	0	1 (1.1)	0	1

TFR, treatment-free remission.

^a^Deaths were reported >30 days after patients discontinued the study.

^b^New deaths reported since the 96-week data cut-off date.

## Discussion

Overall, the results of this updated analysis provide further support for the durability of TFR following frontline nilotinib treatment in patients with CML-CP. The TFR rate in this updated ENESTfreedom 5-year analysis was 41.6%, similar to earlier reports from the study (48.9% at 1.8 years and 51.6% at 0.9 years) [13, 24]; the lack of substantial change likely results from very few relapses after more than one year following nilotinib discontinuation. These TFR rates are comparable to those reported in other nilotinib studies [26] and also for other TKIs [15, 17, 19].

Almost all patients who experienced loss of MMR following treatment discontinuation readily regained it when nilotinib treatment was re-initiated. Successful TFR requires frequent patient monitoring, and by study design, this testing was less frequent as the TFR phase progressed. The possibility that early molecular relapses may be missed by infrequent monitoring is of concern for clinicians, since patients in this situation would potentially take longer to regain response. However, our results show that treatment re-initiation as soon as the loss of MMR was identified (within 5 weeks) caused *BCR-ABL1*^IS^ transcript levels to fall quickly, highlighting the safety of TFR even with quarterly monitoring. This is supported by a recent modeling study showing that monitoring patients every 2 months in the first 6 months of TFR and every 3 months between 6 and 12 months can minimize delays in relapse detection while reducing the overall number of tests [27].

The AE profile during TFR was consistent with that reported previously for this study and in other TKI studies [28–30]. There was an increase in AEs grouped around musculoskeletal pain after the start of the TFR phase, a phenomenon reported in other studies involving nilotinib [26] as well as other TKIs [12]. However, these events were transient. There was also a considerable increase in all-grade AE frequency observed in the treatment re-initiation phase compared with the consolidation phase. This is consistent with the safety profile of nilotinib and most likely related to increased exposure to the drug; patients in the re-initiation phase had a ≥ 4-fold greater median duration of exposure to nilotinib compared with patients in the consolidation phase. No progression to AP/BC and no CML-related deaths were reported, supporting the notion that TFR does not affect patient outcomes or lead to the development of resistance to TKIs. These results provide further support for the overall safety of the TFR approach with careful molecular monitoring.

As previously mentioned, the average duration of nilotinib treatment for ENESTfreedom patients was ~3.5 years before entering TFR, much shorter than that in other TKI studies [16, 17, 19, 20, 25]. However, the ENESTfreedom approach resulted in good efficacy, with 88/190 patients (46.3%) who started the TFR phase remaining in MR^4.5^ after at least 3 years of nilotinib treatment and 2 years in TFR (5 years in total). To put this result in context, a similar proportion of patients (91/183, 49.7%) with continued 300 mg BID nilotinib treatment in the ENESTnd study remained in MR^4.5^ after 5 years, without any treatment-free periods [30]. Although these two studies are clearly different, the data from ENESTnd serve to better contextualize the results from ENESTfreedom in terms of balancing the possibility of achieving DMR and potential treatment side effects, particularly in patients with underlying cardiovascular risk factors.

Longer treatment duration with nilotinib is associated with increased cumulative rates of MR^4.5^, [30] so it is possible that the proportion of patients maintaining MR^4.5^ in ENESTfreedom after stopping treatment would have increased had the treatment been continued for longer. However, increased exposure to nilotinib is also associated with an increased frequency of CVEs [30]. While 6 CVEs (3.2%) were reported during the first 2 years of TFR (2 years of TFR after 3 years of nilotinib treatment, totaling 5 years), and 10 CVEs (5.3%) were reported during the first 5 years of TFR for 190 patients in ENESTfreedom, 20/279 patients (7.2%) in ENESTnd reported at least one CVE within the first 5 years of treatment [30]. The true proportion of patients experiencing CVEs is likely underestimated in ENESTnd, as cardiovascular risk awareness with second-generation TKIs was still evolving during the conduct of the study. Cardiovascular risk awareness with second-generation TKIs was more prevalent in clinical practice during the conduct of ENESTfreedom thanks to published guidelines such as the EuropeanLeukemiaNet (ELN) 2016 recommendation on AE management [31] and reviews of the literature [32], possibly leading to more CVEs being reported during the course of the study. There are also notable differences in the patient population of ENESTnd and ENESTfreedom, with a higher median age for ENESTfreedom (55.0 years vs 46–47 years in ENESTnd), as well as a smaller proportion of patients <35 years (23.8% in in the ENESTnd nilotinib 300 mg BID arm vs 8.4% in ENESTfreedom); an ageing population will tend to have an increased proportion of patients with cardiovascular comorbidities. Since the risk of developing CVEs with nilotinib treatment correlates with the presence of cardiovascular risk factors, patients should be screened before starting treatment and provided with appropriate care for comorbidities, particularly during treatment re-initiation, in order to prevent or reduce the development of CVEs.

Overall, it is clear that a careful equilibrium should be reached between increasing the chances of successful TFR and increased burden of AEs: our results suggest that shorter treatment duration with nilotinib before attempting TFR does not substantially decrease the proportion of patients achieving MR^4.5^ and results in decreased frequency of AEs compared with continuous nilotinib treatment.

No reliable marker of TFR success exists, although higher TFR rates have been reported with longer duration of TKI therapy as well as longer duration of molecular response before stopping treatment [33]. Our updated analysis confirmed low Sokal risk score as a potential prognostic indicator of TFR success [24]. Another potential predictor of successful TFR identified was the depth of response at 48 weeks following the start of the TFR phase: patients in MR^4.5^ at this time had numerically greater TFR rates than those in MR^4^ or MMR. These results show the low residual risk of relapse for patients in deep response at the end of the first year of TFR. Furthermore, stable MR^4.5^ during the first year of TFR was also associated with higher TFR rates. The stability of the molecular response has already been proposed as a variable influencing TFR success [17], but not enough data are currently available to establish a definite link. Although the stability of MR^4.5^ is known to be associated with improved patient outcomes [34, 35], to our knowledge, this is the first time it has been reported as a potential predictor of TFR success. However, it should be noted that the number of patients in some of the groups was very small. Interestingly, a low Sokal risk score is associated with the achievement of stable MR^4.5^ in patients treated with imatinib [34], suggesting a link between the two. Although longer duration of response and longer duration of nilotinib treatment before entry into TFR were also associated with numerically higher TFR rates, these were not strong predictors of TFR success in our analysis. Furthermore, since both of these rely on factors outside the study design and protocol, these results should be approached with caution.

In summary, our findings show sustained long-term high TFR rates in patients with CML-CP treated upfront with nilotinib, highlighting the effectiveness and safety of the approach. First-line nilotinib treatment should be considered for those patients for whom TFR is a treatment goal.

## Supplementary information

Supplementary Table 1

Supplementary Figure 1A

Supplementary Figure 1B
