# Supplementary material for: Treatment-free remission following frontline nilotinib in patients with chronic phase chronic myeloid leukemia: 5-year update of the ENESTfreedom trial
Source: Leukemia. 2021 Mar 11;35(5):1344–55. doi: 10.1038/s41375-021-01205-5 (PMC8102196; doi:10.1038/s41375-021-01205-5)
Supplement: Supplementary file 1 — Supplementary Table 1 [file 41375_2021_1205_MOESM1_ESM.docx]

**Supplementary Table 1. Duration of exposure to nilotinib by study phase.** Duration of exposure (in weeks) was calculated as (date of last dose – date of first dose + 1 day)/7. Duration of non-exposure for TFR and TFR-2 phases was calculated as duration of off-treatment phase.

| **Study phase** | **Consolidation phase N=215** | **TFR N=190** | **Re-initiation phase N=91** |
| --- | --- | --- | --- |
| **Median duration of exposure, weeks (range)** | 52.14  (8.6–57.6) | NA | 224.0  (5.0–287.7) |
| **Median duration of non-exposure, weeks (range)** | NA | 75.86  (7.6–303.0) | NA |

NA, not applicable; TFR, treatment-free remission.
