# Supplementary material for: Treatment-free remission following frontline nilotinib in patients with chronic phase chronic myeloid leukemia: 5-year update of the ENESTfreedom trial
Source: Leukemia. 2021 Mar 11;35(5):1344–55. doi: 10.1038/s41375-021-01205-5 (PMC8102196; doi:10.1038/s41375-021-01205-5)

Supplementary Figure 1. A.
Cumulative incidence of first musculoskeletal pain event in the TFR phase.

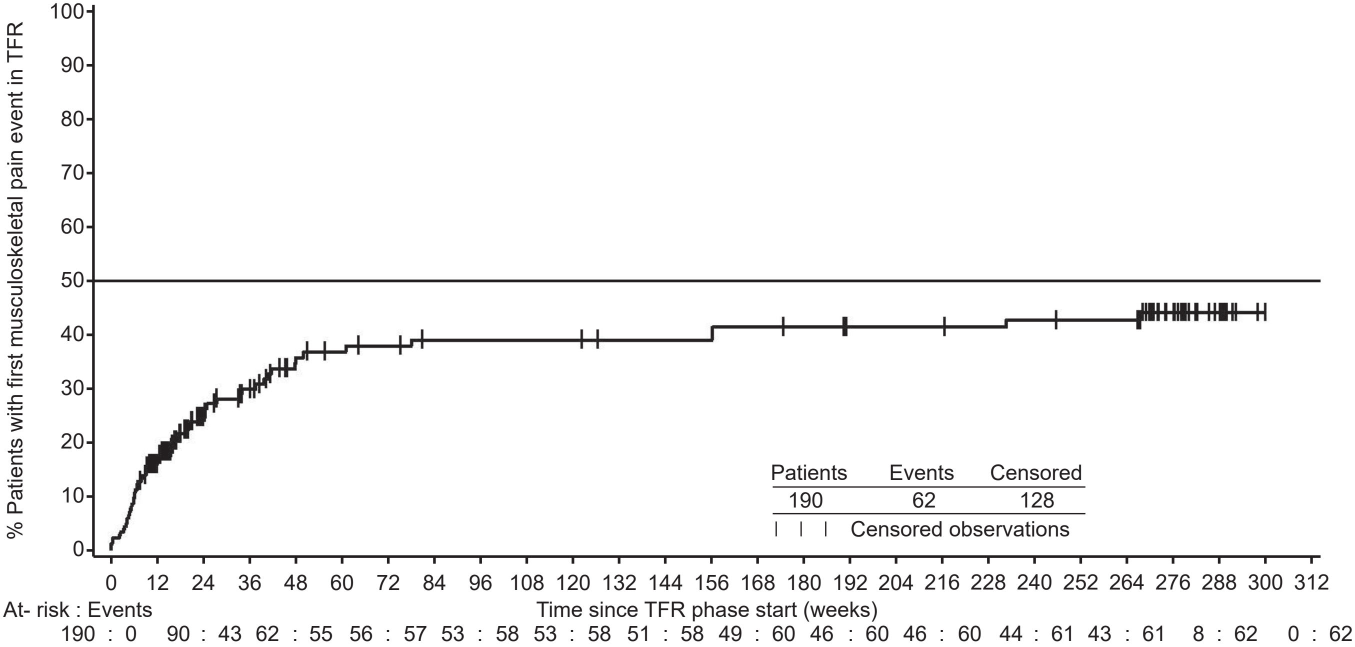

Supplement: Supplementary file 2 — Supplementary Figure 1A [file 41375_2021_1205_MOESM2_ESM.pdf]
